# Book Reviews

**Published:** 1895-12

**Authors:** 


### *BOOK REVIEWS.*

---

The Pathology and Treatment of Venereal Diseases. By Robert W. Taylor, A.M., M.D., Clinical Professor of Venereal Diseases in the College of Physicians and Surgeons, New York. In one very handsome octavo volume of 1002 pages, with 230 engravings and seven colored plates. Cloth, $5.50; leather, $6.50. Philadelphia: Lea Brothers & Co., Publishers. 1895.

For a number of years practitioners were familiar with the classic work of Bumstead and Taylor on venereal diseases in its five successive editions. While the work before us is in a certain sense the successor of these former editions, it has been made a new book in accordance with modern pathology and present-day methods of treatment. Dr. Taylor has entirely rewritten the whole subject, and now presents the profession with a treatise that is clear, practical, and scholarly, and right up to date.

In the Introduction the author gives a concise history of the venereal diseases, with special reference to chancroid and syphilis and their recognition as distinct and separate pathological entities. He also here teaches the non-specific nature of chancroid—that it may originate *de novo* from infection with the products of simple inflammation. In the treatment of gonorrhea the author takes a conservative stand with respect to the thousand and one methods and remedies lately proposed; he finds that the time-honored methods of treatment, if patiently and intelligently carried out, will yield results fully equal to those which can be gotten by many of the trumped-up devices and solutions of recent date.

The best portion of this work is the section on Syphilis, comprising about half of the volume. Every phase and manifestation of the disease are here presented and discussed in a manner which could scarcely be improved upon. One hundred pages are devoted to a consideration of the "General Methodical Treatment of Syphilis," a chapter which gives an admirable outline of the management of syphilitic patients—moral, hygienic, and medicinal.

Dr. Taylor's book deserves a high place among works of this class. One work on this subject is a necessity in any library. There is none better than this.

TWENTIETH CENTURY PRACTICE. An International Encyclopedia of Modern Medical Science. By leading authorities of Europe and America. Edited by Thomas L. Stedman, M.D., New York City. In Twenty Volumes. Volume IV. Diseases of the Vascular System and Thyroid Gland. New York: William Wood and Company. 1895.

The fourth volume of this popular work begins with the Diseases of the Heart and Pericardium. This article is from the pen of Dr. Jas. T. Whittaker of Cincinnati, which is sufficient guarantee of its character and excellence. It occupies about 450 pages, of which the diseases of the pericardium take about sixty, and diseases of the heart itself, with the endocardium, the remainder. The section on Neuroses of the Heart (Neurasthenia, Palpitation, Tachycardia, Bradycardia, Arythmia, and Angina Pectoris) is the best discussion of these conditions which we have seen. Diseases of the Blood-vessels are described by Dr. A. Ernest Sansom of London, a well-known authority on these affections. Dr. Bertrand Dawson, also of London, next describes the Diseases of the Lymphatic Vessels.

The section on Diseases of the Thyroid Gland, including Myxedema, Cretinism, and Goitre, closes the volume, and is of special interest at this time. It occupies 134 pages. Exophthalmic goitre is very properly included in this section, instead of among the neuroses of the heart. This section is written by Dr. George Murray of Newcastle-on-Tyne, to whom we owe the introduction of the thyroid gland treatment for myxedema, cretinism, etc. This is the first time, we understand, that the author has presented this subject in a complete and systematic form. This article is well illustrated, showing striking results of this treatment.

The contents of this volume are in thorough keeping with the high excellence established by its predecessors.

---

PHYSICIANS' VISITING LIST FOR 1896. P. Blakiston, Son & Co., 1012 Walnut street, Philadelphia. Price (twenty-five patients per week), $1.00.

This is the forty-fifth year of publication of this popular Visiting List. Some improvements have been made over previous issues, which make the book more convenient and serviceable. It will be found to fulfill all the requirements of a good visiting list.

PRACTICAL URANALYSIS AND URINARY DIAGNOSIS: A Manual for the Use of Physicians, Surgeons, and Students. By Charles W. Purdy, M.D., Professor of Urology and Urinary Diagnosis at the Chicago Post-Graduate Medical School. Second revised Edition. With Numerous Illustrations, including Photo-Engravings and Colored Plates. $2.50 net. Philadelphia: The F. A. Davis Co., Publishers, 1914 and 1916 Cherry street.

The demand for a second edition of this work within a few months after its first appearance is evidence of the general favor which this book has received. It has not been thought necessary to make many changes in the original volume, though a few additional colored plates have been put in. We need only say now, as we said in our former review of it, that for a practical guide to the methods of urinary analysis and diagnosis there is probably nothing better. We think *urinalysis* a better spelling than the one adopted by the author.

---

ELECTRO-THERAPEUTICAL PRACTICE. By Chas. S. Neiswanger, Ph.G., Professor of Electro-Physics, Post-Graduate Medical School of Chicago. Price $2.00. Published by E. H. Colegrove & Co., 52 Randolph street, Chicago.

This little book is intended to be a ready-reference guide for physicians in the use of electricity. The author does not teach that electricity is the universal specific, and indeed makes no extravagant claims for this therapeutic agent; he only suggests the proper methods of supplying it in such cases as, in the judgment of the physician, seem to make the use of it desirable. Those who desire some help along this line will find this little volume very useful.

---

THE MEDICAL RECORD VISITING LIST. For 1896. Revised edition. William Wood & Co., 45–47 East 10th street, New York.

This Visiting List has enjoyed a well-deserved popularity with the profession for many years. The issue for 1896 has been prepared so as to make it as nearly as possible an ideal memorandum book for the physician in his work. Doctors will find it eminently satisfactory. It contains the usual tables of doses, antidotes, emergency information, etc.

